# Critical appraisal of the role of serum albumin in cardiovascular disease

**DOI:** 10.1186/s40364-017-0111-x

**Published:** 2017-11-10

**Authors:** Shih-Chieh Chien, Chun-Yen Chen, Chao-Feng Lin, Hung-I Yeh

**Affiliations:** 1 Department of Critical Care Medicine, MacKay Memorial Hospital, No. 92, Sec. 2, Zhongshan N. Rd, Taipei City, 10449 Taiwan, Republic of China; 2 Cardiovascular Division, Department of Internal Medicine, MacKay Memorial Hospital, Mackay Medical College, No. 92, Sec. 2, Zhongshan N. Rd, Taipei City, 10449 Taiwan, Republic of China

**Keywords:** Serum albumin, Cardiovascular disease, Prognosis, Literature review

## Abstract

Concentration of serum albumin (SA), a multifunctional circulatory protein, is influenced by several factors, including its synthesis rate, catabolism rate, extravascular distribution, and exogenous loss. Moreover, both nutritional status and systemic inflammation affect the synthesis of SA. Determining SA concentration aids in risk prediction in various clinical settings. It is of interest to understand the prognostic value of SA in the full spectrum of cardiovascular disease (CVD) in the era of newly developed pharmacological and interventional treatments. Proper interpretation of SA in addition to established risk factors potentially provides a better risk discrimination and thereby presents an option to modify therapeutic strategies accordingly. In this narrative review, we summarize the basic features of SA and its associated physiological functions contributing to its prognostic impacts on CVD. Finally, we discuss the prognostic role of SA in CVDs based on existing evidence.

## Background

The properties and role of albumin have been explored for more than five centuries, since it was first precipitated from urine by Paracelsus [1]. Nowadays, we already have clarity on much of the information about albumin from studies carried out. Serum albumin (SA), the most abundant circulatory protein, is associated with several vital physiological functions, such as maintaining oncotic pressure and microvascular integrity, regulating metabolic and vascular functions, providing binding ligands for substances, antioxidant activities, and anticoagulant effects. Furthermore, SA is inversely related to inflammatory processes by modulating neutrophil adhesion and cell signaling moieties [2, 3].

SA is traditionally regarded as a biomarker for reliable risk prediction in various clinical settings. An increased risk in all-cause mortality and cardiovascular (CV) mortality has been shown to be associated with low SA concentration [4]. In the current era, innovative revascularization procedures and emerging medical therapies have altered the clinical features of cardiovascular disease (CVD) and its prognosis. Hence, identifying new biomarkers and re-evaluating the significance of traditional risk factors are important [5]. It is also interesting to understand the prognostic value of SA in the full spectrum of CVD so that the physicians can employ SA for risk prediction among selected populations and thereby offer an optimal therapeutic strategy accordingly.

This article reviews the basic concepts and the proposed functions of albumin, focusing on the link between SA and CVD based on existing evidence.

## Structure and biochemistry of albumin

Albumin is a member of the albumin superfamily, which also includes the transport proteins α-fetoprotein, vitamin D -binding protein (Gc-globulin), and afamin (α-albumin). Albumin is an aglycosylated, negatively charged protein consisting of 585 amino acids forming a single polypeptide chain of molecular weight 66.5 kDa [6, 7]. The chain is composed of abundant charged residues such as lysine and aspartic acid and has no prosthetic groups or carbohydrate attached to it. Structurally, albumin has a heart shape in x-ray crystallography (measuring 80 × 30 Å) but in solution its conformation is ellipsoid [3]. The mature, circulating molecule is arranged in a series of α-helices, stabilized by 17 disulfide bonds, and comprises three homologous domains (I–III). Each of these domains has two subdomains (A and B) composed of 4 and 6 α-helices, respectively. Albumin in solution is characterized by a flexible texture, owing to the presence of disulfide bonds between domains that confer ability to change between different conformational states. One unbound cysteine residue (Cys-34) in subdomain IA is a redox active thiol (−SH) group. The thiol moiety is capable of thiolation (HSA-S-R) and nitrosylation (HSA-S-NO) as well as binding of substances [7].

## Metabolism and distribution of albumin

The total albumin pool is about 3.5–5 g/kg body weight, of which 40–45% is present in the intravascular space and the remaining is present in the interstitial space [2]. The reference range for albumin concentrations in serum is approximately 3.5–5.0 g/dL and the cases with the concentration less than 3.5 g/dL is usually referred as hypoalbuminemia [8]. Albumin changes from intravascular to extravascular compartment at a rate of 5%/hour and the degradation corresponds to 5% of the daily whole-body protein turnover [2, 3] The transcapillary escape rate is increased in a variety of diseases [9]. In general, SA excretion via urine is minimal. This could be explained from its size and charge as well as its active reabsorption in renal tubuli [10]. Albumin is degraded into amino acids in all parts of the body. The biological half-life of SA is almost three weeks [10]. The amount of albumin metabolized, approximately 10% of the concentration per day under normal physiological conditions [9].

Synthesis of albumin occurs in the liver and is influenced by several factors, including diet and nutrition, colloidal oncotic pressure, exposure to hormones, and disease states (Table 1). Albumin synthesis may be suppressed by fasting or consumption of a protein-deficient diet [11], exogenous supply of substances raising colloidal oncotic pressure [12, 13], and comorbid diabetes or hepatic disease [14, 15]. Conversely, albumin synthesis increase by consumption of a high-protein (amino acid) diet [11] and exposure to growth hormones, corticosteroids as well as insulin [14, 16, 17].

**Table 1:** Factors influencing albumin synthesis

Factors that increase synthesis	Factors that decrease synthesis
High-protein diet [11]	Protein-deficient diet [11]
High calories [11]	Fasting [11]
COP↓ [12, 13]	COP↑ [12, 13]
Growth hormone [15]	Diabetes [14]
Corticosteroids [16]	Hepatic disease [17]
Insulin [14]	Sepsis, trauma [2]

*Abbreviation*: *COP* colloidal oncotic pressure

## Biological functions of albumin

### Colloid oncotic pressure

Albumin has lower molecular weight than the average of serum globulins but its concentration is higher conferring major osmotic significance, contributing to 80% of total plasma colloid oncotic pressure. The osmotic pressure from SA concentration alone accounts for approximately 60% of the total oncotic pressure and the remaining 40% is attributed to the negative charges surrounding the protein, which attract and retain cations, particularly Na + in the vascular compartment. This effect is so called the Gibbs–Donnan effect, which retains water and enhances the oncotic pressure [2].

### Binding capacities of albumin

The presence of a net charge and the ligand-binding sites enable SA to bind to and transport various substances, either endogenous or exogenous, such as inorganic ions, trace elements, vitamins, fatty acids, bilirubin, hormones, thyroxine, steroids, and drugs. These important binding sites for different substances have been well studied [7]. The binding capacities are influenced by several factors such as SA concentration, comorbidities, or presence of competitive compounds [2].

### Blood viscosity and vascular function

An animal study showed that lack of albumin reduced erythrocyte deformability and subsequently increased blood viscosity [18]. This phenomenon of low SA and high blood viscosity is also observed in patients with nephrotic syndrome [19]. Moreover, SA is suggested to maintain the permeability of the capillary membrane [20]. Furthermore, it is proposed that hypoalbuminemia impairs the vasodilatory response to nitric oxide (NO) [21].

### Antioxidant effects

Oxidative stress, due to reactive oxygen species (ROS) or reactive nitrogen species (RNS), can inflict damage on molecules, leading to the accumulation of toxic end products causing cellular dysfunction. This has been implicated in the pathogenesis of inflammation and atherosclerosis. Physiologically, SA contains abundant thiol groups, accounting for 80% of the total thiols in plasma scavenging ROS or RNS [3]. Moreover, a number of albumin-bound substances, such as NO and bilirubin, possess antioxidant properties, contributing to additional protection from oxidative stress [22, 23].

### Anticoagulant effects

Investigations have demonstrated that low SA concentration is related to attenuated fibrinolysis [24]. The bioavailability of prostacyclin (PGI2), a naturally occurring vasodilator and a potent inhibitor of platelet aggregation, is dependent on the albumin concentration [25]. SA is also proposed to have a heparin-like activity. This effect is possibly explained by the similarities in structure and net charge with heparin [2].

### Inflammatory responses of serum albumin

The activation of inflammatory response leads to a relevant change in plasma proteins, cytokines, and complements. SA is a negative-phase protein whose concentration decreases in response to inflammation. Although the mechanisms are not completely clear, it is presumed that a large proportion of amino acid is utilized to synthesize positive-phase proteins rather than albumin in the liver during an inflammatory response [26]. This hypothesis is supported by the investigations on sialylated glycoproteins in serum. Some of the sialylated glycoproteins rapidly increase in response to the onset of inflammation and are regarded as an index of acute phase response [27].

### Regulating cholesterol transport

Although albumin does not have specific binding sites for cholesterol, it is considered to be a regulator of cholesterol transport. Aqueous transfer is an important mechanism contributing to cellular cholesterol efflux and SA acts as a shuttle, enhancing aqueous diffusion. [28] Moreover, SA is showed specific binding to vesicles which contained cholesterol [29]. Study showed that cholesterol efflux reduced approximately 40% from fibroblasts in the absence of plasma albumin by immunoaffinity chromatography [30]. It is proposed that cholesterol flux between numerous cholesterol pools would be enhanced by SA, thereby facilitating the restoration of steady-state levels as cholesterol is metabolized [28]. This effect possibly aids in explaining the relationship between SA and CVD.

## Serum albumin and prognosis in cardiovascular disease

**Fig. 1:**
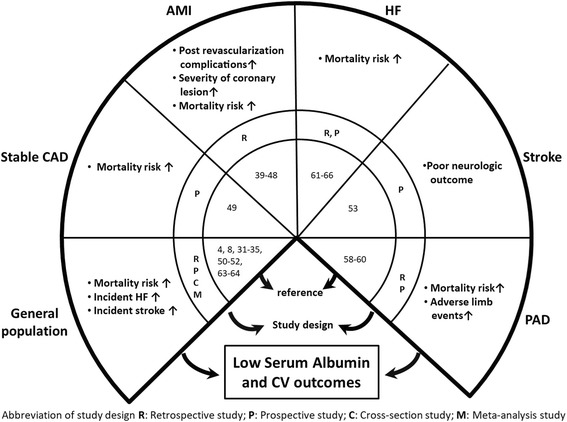
Serum albumin and prognosis across the cardiovascular continuum. Abbreviation: CAD: coronary artery disease; AMI: acute myocardial infarction, HF: heart failure; PAD: peripheral arterial disease

**Fig. 2:**
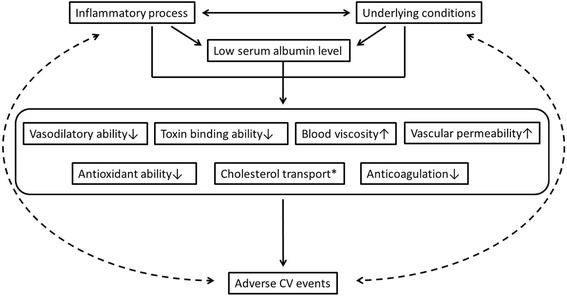
Proposed interaction between serum albumin and CV outcomes. Abbreviation: CV: Cardiovascular. * Serum albumin is proposed to enhance cholesterol transport between numerous cholesterol pools, facilitating the restoration of steady-state levels as cholesterol is metabolized [28]

SA concentration serves as a reliable prognostic factor for CVD since a serendipitous finding in 1989 [8]. Numerous studies were designed and performed with the purpose of explaining the mechanisms, verifying the relationship, and extending the applications to relevant fields (Fig. 1) (Table 2). In general, the association of low SA concentration and adverse outcomes is thought to be multifactorial. All the above mentioned biological functions of SA, including the maintenance of vascular integrity [20], vasodilatory effects [21], binding ability for toxins [22, 23], regulating cholesterol transport [28–30], anticoagulant effects [2, 24, 25], and antioxidant capacities [2, 3, 22, 23], are implicated in the pathogenesis. Moreover, reduced SA concentration can also be the result of inflammatory conditions [26] or inadequate nutritional intake [11], rendering potential implications on the prognosis of CVD (Fig. 2).

**Table 2:** Clinical studies investigating the relation between serum albumin and cardiovascular disease

Study	Year	Number	Characteristics of patients	Albumin cut-off value	Outcomes
Coronary artery disease (CAD)					
Hartopo et al. [42]	2010	82	ACS	< 3.5 g/dL	↑In-hospital adverse event^a^
Bhamidipati et al. [43]	2011	2794	CAD undergoing CABG	< 3 g/dL	↑Mortality and post-operative complications
Oduncu et al. [39]	2013	1706	STEMI undergoing primary PCI	< 3.5 g/dlL	↑3.5-year all mortality and advanced HF
Sujino et al. [40]	2015	62	Age ≧ 85 years, STEMI	Continuous variable	↑In-hospital mortality
Murat et al. [46]	2015	890	ACS undergoing PCI	Continuous variable	↑Contrast induced acute kidney injury and in-hospital mortality
Kurtul et al. [47]	2015	536	STEMI undergoing PCI	< 3.75 g/dL	↑Risk of no-reflow after PCI
Plakht et al. [41]	2016	8750	AMI	categorize albumin level by 3.4, 3.7, 3.9, 4.1 g/dL	↑10-year all mortality
Kurtul et al. [45]	2016	1303	ACS undergoing coronary angiography	< 3.3 g/dL	↑SYNTAX score and in-hospital mortality
Celik et al. [48]	2016	341	PCI with a BMS	< 3.81 g/dL	↑Risk of in-stent restenosis after PCI
Wada et al. [44]	2017	2860	CAD undergoing PCI	3.8 g/dL; 4.1 g/dL	↑MACE and 10-year all mortality^b^
Chien et al. [49]	2017	734	Stable CAD	< 3.5 g/dL	↑1.5-year MACE and mortality^c^
Ischemic stroke					
Gillum et al. [50]	1994	4897	White men aged 65–74 years; Blacks aged 45–74 years	< 4.2 vs > 4.4 g/dL	↑14-year stroke incidence and stroke mortality
Dziedzic et al. [53]	2004	759	Acute ischemic stroke	< 4.9 g/dL	Poor 3-month neurologic outcome^d^
Hostmark et al. [52]	2006	5071	30–75 years	< 4.7 g/dL	↑(self-reported) Stroke incidence
Xu et al. [51]	2014	2986	>40 years old	< 4.2 vs > 4.6 g/dL	↑12-year stroke incidence
Peripheral Arterial Disease (PAD)					
O’Hare et al. [56]	2002	14,427	Hemodialysis patients	Continuous variable	↑PAD incidence
Beddhu et al. [57]	2002	1411	Hemodialysis patients	< 3.6 g/dL	↑PAD incidence
Schillinger et al. [60]	2004	702	PAD patients with less than 2 traditional risk factors	< 3.85 g/dL	↑1-year MACE^f^
Ishii et al. [59]	2013	450	Hemodialysis patients undergoing endovascular therapy	< 3.6 g/dL	↑3-year major adverse limb event^e^
Tsai et al. [58]	2015	444	Hemodialysis patients	Continuous variable	↑4-year all or CV mortality
Heart failure (HF)					
Horwich et al. [61]	2008	1726	Systolic HF	≤ 3.4 g/dl	↑1- and 5- year all mortality ↑Urgent heart transplant
Kinugasa et al. [63]	2009	349	Age ≧ 65, Acute HF	< 3.2 g/dL	↑In-hospital mortality
Uthamalingam et al. [64]	2010	438	Acute, systolic HF	< 3.4 g/dL	↑1-year cardiac mortality
Gopal et al. [67]	2010	2907	Aged 70–79 years without HF	< 4.0 g/dl	↑6-year HF risk but the risk declined annually
Filippatos et al. [68]	2011	5450	Aged ≥65 years, without HF	≤ 3.5 mg/dL	↑10-year HF incidence
Liu et al. [62]	2012	576	Acute HF, preserved EF	≤ 3.4 g/dl	↑1-year all mortality
Polat et al. [65]	2014	135	Acute, systolic HF	< 3.1 g/dL	↑1-year mortality
Bonilla-Palomas et al. [66]	2014	362	Acute HF	≤ 3.4 g/dL	↑In-hospital and 1-year all mortality

*Abbreviation*: *STEMI* ST-segment elevation myocardial infarction, *HF* heart failure, *PCI* percutaneous coronary intervention, *AMI* acute myocardial infarction, *ACS* acute coronary syndrome, *CAD* coronary artery disease, *CABG* coronary artery bypass graft, *CV* cardiovascular, *MACE* major adverse cardiac event, *BMS* bare-metal stent, *PAD* peripheral arterial disease, *EF* left ventricular ejection fraction

Continuous variable is addressed if studies analyzed the influence of serum albumin with a manner of continuous variable instead of a definite cut-off value

^a^In-hospital adverse events include mortality, acute heart failure, cardiogenic shock, and reinfarction

^b^MACE include nonfatal myocardial infarction and mortality

^c^MACE include nonfatal myocardial infarction, nonfatal stroke and cardiovascular mortality

^d^Poor neurologic outcome was defined as modified Rankin Scale >3 or mortality

^e^Major adverse limb event was defined as a composite of target lesion revascularization, amputation and all-cause mortality

^f^MACE include a composite of nonfatal MI, coronary revascularization, and all-cause mortality

### Albumin and mortality in the general population

The majority of studies supported that lower SA concentration is associated with an increased risk of mortality. Phillips et al. first conducted a population-based, prospective cohort study (British Regional Heart Study), involving 7735 middle-aged men with or without a preexisting disease to evaluate the correlation between SA concentration and mortality. As a result of an average 9.2 years of follow-up, SA concentration was found to be inversely correlated with all-cause mortality, CV mortality, cancer mortality in both the groups with or without a preexisting disease [8]. This result was further supported by another longitudinal analysis from National Health and Nutrition Examination Survey Epidemiologic Follow-up Study (NHEFS). SA concentration of ≥4.5 g/dL was associated with a reduced risk of all-cause mortality (RR: 0.73, 95% CI: 0.62–0.85 for men and RR: 0.71, 95% CI: 0.59–0.85 for women), CV mortality (RR: 0.69, 95% CI: 0.55–0.85 for men and RR: 0.74, 95% CI: 0.58–0.96 for women) and non-CV mortality (RR: 0.77, 95% CI: 0.61–0.97 for men and RR: 0.66, 95% CI: 0.50–0.88 for women) [31] Kuller et al. reported that SA concentration could be an independent predictor of mortality and morbidity in middle-aged men with a high risk of coronary heart events. The association was stronger for coronary heart disease deaths than for nonfatal myocardial infarction (MI) [32]. In the Paris Health Study, there was a significant inverse association observed between SA concentration and all-cause mortality in men, but not in women [33]. This result was contrary to that of the Framingham Offspring Study, which reported an inverse relation between SA concentration and mortality in women but not in men [34]. Results from a meta-analysis, including 8 prospective studies, have shown that subjects with SA levels in the bottom third (estimated 3.8 g/dL) have a 1.9-fold increased risk for all-cause mortality and 2.0-fold increased risk for CV mortality, compared with those in the top third (estimated 4.2 g/dL) [4]. All the aforementioned studies were published before or around 2000. In a recent Japanese cohort study, low SA concentration independently predicted all-cause and cause-specific (cancer, infection and CVD) mortality after a long-term (15 years) follow-up [35].

### Albumin and coronary heart events in the general population

Whether SA concentration could predict nonfatal coronary heart events is an interesting but controversial issue. In the Multiple Risk Factor Intervention trial and the Framingham Offspring study, there was a significant inverse relationship between SA concentration and risk of MI [32, 34]. In the Atherosclerosis Risk In Communities (ARIC) Study, low SA concentration was only associated with incident MI in individuals who were current smokers [36]. Moreover, low SA did not provide adequate predictive power for incident coronary heart events in the older populations (aged 65–74 years) in NHEFS [31]. From a prospective study on the elderly (aged 65 years and older), low SA concentration was identified with a high risk for coronary heart events in women but not in men [37]. In Zutphen Elderly Study, SA only predicted the incidences of coronary heart events among men with elevated total cholesterol [38]. Thus, the risk discrimination of incident coronary heart events using SA concentration is not consistent and varies with different designs of studies. It is possible that low SA concentration does have a direct causative role for incident coronary heart events but could be an indicator of an underlying condition [34]. Similar to the previous section, all the data reviewed here were derived from studies published before or around 2000.

### Albumin and prognosis in coronary artery disease

In the presence of effective pharmacological and interventional treatments for coronary artery disease (CAD) after the millennium, the prognostic value of SA has been emphasized further. Among individuals with an acute phase of CAD, SA is also reported as a strong prognostic factor [39–41]. Hartopo et al. evaluated small number patients with acute coronary syndrome (ACS) from a single center. Hypoalbuminemia (< 3.5 g/dL) measured upon admission was associated with in-hospital adverse events, including death, acute heart failure, cardiogenic shock, and reinfarction. This association, however, is not significant by adjusting other risk factors (OR: 2.8, 95% CI: 0.7–9.5) [42] Oduncu et al. retrospectively analyzed 1706 patients with ST-segment elevation myocardial infarction (STEMI) treated with primary percutaneous coronary intervention (PCI), and reported that hypoalbuminemia is associated with both in-hospital mortality and long-term mortality, with a median follow-up of 40 months (23.3 vs. 8.4%, *P* < 0.001). In a multivariate model, hypoalbuminemia independently predicted long-term mortality (HR: 2.98, 95% CI: 1.35–6.58) [39]. The importance of SA in the older patients with STEMI was also demonstrated [40] Plakht et al. categorized SA concentration more delicately in patients with acute MI. They found that SA concentration upon admission provided clear risk discrimination for 10-year mortality [41].

Among patients undergoing isolated coronary artery bypass graft, higher preoperative SA concentration reduced the 30-day and/or in-hospital mortality and major complications [43]. Similar prognostic impacts were also reported among those undergoing PCI [44]. SA could be used as an important indicator of angiographic finding or adverse events associated with PCI. ACS patients with hypoalbuminemia had higher frequencies of multivessel disease, a higher thrombus burden, and a longer lesion length [39]. Low SA concentration(< 3.65 g/dL) was proposed as an independent predictor of high SYNTAX score (≥33) and both factors, SA and SYNTAX score, were linked with the outcomes [45]. Moreover, SA concentration on admission is inversely associated with post-PCI contrast-induced acute kidney injury in patients with acute coronary syndrome [46]. Furthermore, SA concentration is an important predictor of no-reflow phenomenon following primary PCI and in-stent restenosis rate after bare-metal stent implantation [47, 48].

We have recently reported the results of hypoalbuminemia and adverse CV outcomes in the Biosignature study, which enrolled CAD patients in stable condition at baseline prospectively. Patients with hypoalbuminemia had an increased risk of 1.5-year in major adverse CV events and in all-cause mortality (major adverse CV events, HR: 3.68, 95% CI: 1.03–13.19; all-cause mortality, HR: 6.81, 95% CI: 1.01–45.62) [49]. This study unequivocally confirms the important association between SA and individuals with chronic stable CAD.

### Albumin and prognosis in ischemic stroke

Data from the First National Health and Nutrition Examination Survey (NHANES I) Epidemiologic Follow-up Study suggested that SA concentration < 4.2 g/dL was associated with an increased risk of stroke incidence and death compared with SA >4.4 g/dL in middle-aged white men and black men [50]. The inverse relation of reduced SA and incident stroke was also observed in two studies (the Northern Manhattan Study and the cross-sectional Norwegian Oslo Health Study) [51, 52]. Dziedzic et al. investigated 759 patients with acute ischemic stroke. The reduced risk of poor outcome, defined as modified Rankin Scale >3, or death was observed within the upper quartile of SA (OR: 0.43, 95% CI: 0.26–0.7) [53]. Despite the apparent relations between higher SA concentration and better outcome from epidemiologic evidence, active therapy with albumin solution in acute ischemic stroke did not improve the outcome. This was true also for intracerebral hemorrhage and pulmonary edema where increased risk rates were identified [54, 55].

### Albumin and prognosis in peripheral arterial disease

A cross-section investigation suggested SA concentration is also inversely associated with the incidence of peripheral artery disease (PAD) among patients undergoing hemodialysis [56]. Moreover, data from the HEMO study, enrolling 1411 patients undergoing hemodialysis, suggested that the risk for CVD linearly increased as albumin level decreased in patients with SA concentration ≥ 3.6 g/dL. The odds ratio with each 1 g/dL increase in SA decreased by 61% for PAD, 68% for CAD, 67% for CVD, and 77% for all atherosclerotic diseases [57].

Low SA concentration is associated with an increased risk of all-cause mortality among hemodialysis patients with PAD in a single center retrospective study [58]. It was also proposed as a single or add-on predictor of major adverse limb events, a composite of target lesion revascularization, amputation and all-cause mortality in hemodialysis patients undergoing endovascular intervention [59]. However, a prospective study enrolling angiographically proven PAD patients suggested that the prognostic value of SA in major adverse cardiac events, a composite of MI, coronary revascularization, and all-cause mortality was limited to the subjects who had only two traditional risk factors or less [60]. The prognostic effect of SA in PAD is attenuated when the severity or the complexity of comorbidities increases, contributing to a major impact on the outcomes.

### Albumin and prognosis in heart failure

The development of heart failure (HF) can be the result of a sole cardiac disorder or, rather more often, the results from other CVD and impaired cardiac function and structure. Hence, the relationship between SA and HF is liable to be considered here. Hypoalbuminemia is prevalent in HF patients, in approximately one-third of those in chronic state and in up to half of all hospitalized patients [61, 62]. Hypoalbuminemia (≤ 3.2 g/dL) is an important predictor of in-hospital mortality among elderly patients who were admitted for acute HF [63]. Hypoalbuminemia appears to be reliable in predicting mortality among individuals with HF and a reduced ejection fraction (< 40%), both in acute [64–66] and chronic states [61]. However, results from individuals of HF and preserved ejection fraction (HFpEF) in two retrospective studies differed. Uthamalingam et al. evaluated 37 HFpEF patients who were admitted for acute decompensated HF; hypoalbuminemia was not found to be clearly associated with 1-year mortality [64]. In another study conducted by Liu et al. enrolling more participants, i. e. 611 individuals consecutively admitted for acute decompensated HF with preserved ejection fraction, hypoalbuminemia was found to be useful in predicting 1-year all-cause mortality after adjusting the baseline factors [62].

SA and HF risk association was investigated in elderly patients. A community-based cohort study investigated 2907 healthy individuals aged between 70 and 79. Those with low baseline SA concentrations were at risk for developing HF, but this risk declined over time [67]. In the Cardiovascular Health Study, enrolling 5450 healthy individuals aged ≥65 years, hypoalbuminemia was found to be significantly associated with incident HF in 10 years by using propensity matched analysis [68]. Since the established study reported a significant association between echocardiographic parameters and HF, the impact of SA on cardiac dysfunction should be investigated [69]. This would not only elucidate the link between SA and HF but also aid early detection of subclinical cardiac dysfunction in healthy individuals.

## Conclusions

The measurement of SA is widely available and popular in current medical institutes because of its perceived reliability and low cost. Since SA implicates several important physiological functions, the determined SA concentration reflects physical or healthy status and thereby predicts the prognosis. It is clear that an improvement in risk-specific approaches, which can provide robust and accurate risk prediction, are needed to curb the ongoing burden of CVD [70]. We have to be acquainted with applications of albumin in comprehensive CV fields. Tailored therapies, such as nutritional intervention and direct albumin administration, in individuals with low SA concentration, considered as high-risk groups, should be investigated with randomized placebo-controlled trials to understand its clinical efficacy and safety [71]. In addition, it is warranted to continue exploring the additional roles of albumin in CVD.

## Abbreviations

ARIC: Atherosclerosis Risk In Communities Study
CAD: Coronary artery disease
CV: Cardiovascular
CVD: Cardiovascular disease
HF: Heart failure
HFpEF: Heart failure with preserved ejection fraction
MI: Myocardial infarction
NHEFS: National Health and Nutrition Examination Survey Epidemiologic Follow-up Study
NO: Nitric oxide
PAD: Peripheral artery disease
PCI: Percutaneous coronary intervention
RNS: Reactive nitrogen species
ROS: Reactive oxygen species
